# In-Class Cycling to Augment College Student Academic Performance and Reduce Physical Inactivity: Results from an RCT

**DOI:** 10.3390/ijerph14111343

**Published:** 2017-11-04

**Authors:** Lanae Joubert, Matthew Kilgas, Alexandrea Riley, Yuba Gautam, Lars Donath, Scott Drum

**Affiliations:** 1 School of Health and Human Performance, Northern Michigan University, Marquette , MI 49855, USA; aholley@nmu.edu (A.R.); ygautam@nmu.edu (Y.G.); sdrum@nmu.edu (S.D.); 2 Department of Kinesiology and Integrative Physiology, Michigan Technological University, Houghton, MI 49931, USA; makilgas@mtu.edu; 3 Institute of Training and Computer Science in Sport, German Sport University Cologne, 50933 Köln, Germany; lars.donath@unibas.ch

**Keywords:** stationary cycling, active workstation, physical activity, academic performance, sedentary behavior, physical inactivity, college students

## Abstract

Most college students sit 14 hours per week on average, excluding sedentary study time. Researchers observing workplace and elementary school settings with active workstations to combat sedentary behavior have shown enhanced cognition without distraction. Until now, incorporating active workstations in college classroom settings remained relatively unexplored. This study’s purpose was to assess academic performance using in-class stationary cycle desks during a semester-long lecture course. Twenty-one college students (19–24 years) enrolled in a lecture course volunteered and were split into traditional sit (SIT) and stationary cycle (CYC) groups randomly, matched on a calculated factor equal to a physical activity (PA) score (0–680) multiplied by grade point average (GPA; 4.0 scale). CYC pedaled a prescribed rate of perceived exertion (RPE) of less than 2 out of 10 during a 50-min lecture, 3 × week for 12 weeks. CYC averaged 42 min, 7.9 miles, and 1.7 RPE during class throughout the semester. No significant differences (*p* > 0.05) were observed between CYC and SIT on in-class test scores or overall course grades. Although statistically insignificant, CYC had higher mean test scores and overall course grades vs. SIT (i.e., B^+^ vs. B, respectively). Low intensity cycling during a college lecture course maintained student academic performance and possibly reduced weekly sedentary behavior time.

## 1. Introduction

According to the World Health Organization, physical activity (PA) is “any bodily movement produced by skeletal muscles that requires energy expenditure” [1]. The same agency declares that a lack of PA is the fourth leading risk factor for global mortality credited with 6% of deaths globally [1]. Physical inactivity has been touted as the biggest public health problem of the 21st century [2]. In the United States (U.S.), about half of the adult (≥18 years) population is not meeting PA recommendations [3]. The same is true of young adults (18–29 years) attending college [4]. Additionally, over the past 15 years, many universities have eliminated the physical education and/or PA requirement [5], which may negatively influence lifelong PA habits. Reduced levels of PA over a lifetime are linked with several cardiometabolic diseases, such as type 2 diabetes, cardiovascular disease, some cancers, and all-cause mortality [6,7]. If greater daily or weekly movement patterns replace some sedentary time, such as incorporating standing, cycling, or stepping into weekly work day schedules, cardio-metabolic risk may be attenuated [8].

Targeting reductions in sedentary behaviors to increase PA and improve health was discussed in a recent publication by Keadle et al. [9], which suggested the need for solution-oriented future research. Current research has focused on younger children’s accumulated school time PA by encouraging active desks (pedaling while sitting at a desk) [10] or eliminating chairs to encourage standing [11]. Interrupting prolonged sedentary time during college years may serve as another solution to this epidemic. To our knowledge, no studies have investigated the use of a lecture-style classroom environment to discourage physical inactivity for college students.

In addition to reducing sedentary time, there is also the potential benefit of enhanced concentration and attention. Previous researchers found increased productivity and concentration while being active in a work setting [12,13]. A study by Mahar et al. [14] showed improved on-task behavior with in-class energizer activities in an elementary school classroom setting. On a college campus, Pilcher and Baker [15] compared undergraduate student test results during two different situations: taking an exam (i.e., logical reasoning section of the Law School Admissions Test-LSAT) while (1) stationary cycling versus (2) traditional stationary sitting position. Even though their results did not suggest an improvement in test scores with cycling, they did suggest that PA in educational settings might help to decrease sedentary behavior without negatively affecting test scores. Their study was an acute PA intervention on test taking performance and it seems reasonable that a longer PA intervention with practiced cycling may lead to improved test scores or overall better academic performance. Perhaps it is possible to enhance both task-performance and PA over a longer period while utilizing a cycle desk. Thus, the purpose of our study was to explore low-level PA using cycle desks for 12 weeks in a university classroom setting and its impact on academic performance in an undergraduate exercise physiology course.

## 2. Materials and Methods

### 2.1. Participants

Students were invited to participate in the study if they were enrolled in an undergraduate exercise physiology course traditionally taught with lecture and lab at a university in the Midwestern U.S. This study complied with the declaration of Helsinki and was approved by the university’s internal review board (#HS14-565). Informed consent was obtained from all study participants before beginning the testing procedures. At the beginning of the semester, 34 students were enrolled in the course and 24 of them (ages 19–24 years; 29% male) volunteered to participate. The first week of the semester, all participants reported their past three-months’ average estimated weekly PA (i.e., duration, intensity, frequency, and mode). A PA score was calculated based on aerobic PA = duration (min/week) × intensity (1 = low, 2 = moderate, 3 = high) × frequency (times per week of aerobic PA) plus (+) estimated minutes per week of general resistance training. Accumulated grade point average (GPA; 4.0 scale) from the previous five semesters was obtained from student degree evaluation records. GPA was then multiplied by the PA score to create a final PA factor for each student. Next, we used the stratified randomization process to equally-match groups based on the PA factor. Groups were assigned to either a traditional desk to sit (SIT) or stationary cycle desk (CYC) to cycle. During the second and third weeks of the semester, familiarization with the stationary cycle desks occurred for CYC only. After two weeks of familiarization, one CYC participant dropped out of the study because of anticipated attention distraction and another two CYC participants dropped the course (unrelated to the study), thus the experimental groups became CYC, n = 9, and SIT, n = 12. The study participants’ self-reported PA score ranged from 0–680 (Table 1). Students not enrolled in the study still attended class and sat in a traditional desk, per normal classroom culture.

### 2.2. Study Design and Context

**Figure 1:**
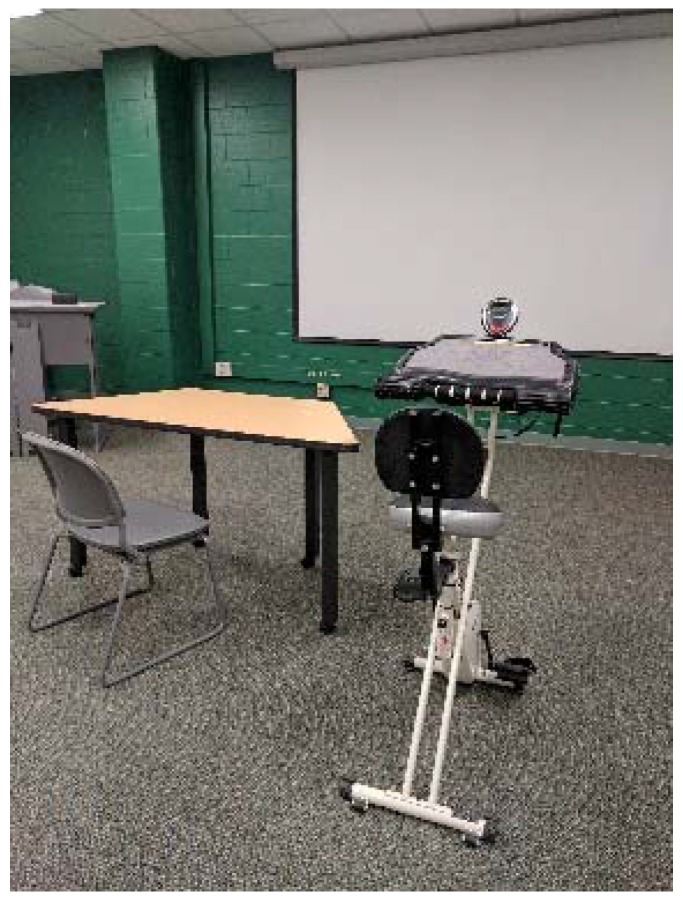
Traditional chair and desk compared to stationary cycle desk (FitDesk, FD Products, Kernersville, NC, USA) in the college lecture classroom.

Stationary cycle desks (FitDesks^®^, FD Products, Kernersville, NC, USA) were randomly placed throughout a lecture classroom to minimize the effect of seating location on learning and attention [16,17]. The traditional desks were standard tables and chairs used in the classroom (Figure 1).

The first week of the 16-week semester was used to inform all students about the study and enroll those interested with informed consent. The participants were told that the researchers were examining general fitness scores at baseline compared to the end of the semester. General fitness scores were derived from these tests: Queen’s college step, timed push-ups and wall sit, sit and reach, and a three-site skinfold measurement. These tests were used to deter their attention from the outcomes of interest (academic performance) to minimize the Hawthorne effect.

#### 2.2.1. Academic Performance Outcomes

Four times throughout the semester, students were given a written examinations (e.g., chapter test) assessing exercise physiology course concepts. Each test consisted of a variety of objective question types: multiple choice, true and false, fill in the blank, short answer, and interpretative graphics. Test I was 90 points total with 41 questions; Test II was 79 points total with 21 questions; Test III was 56 points total and 39 questions; and Test IV was 144 points total, weighted 200 points, with 54 questions. All participants were provided the same tests. The instructor for this course was part of the research team, but blinded when grading the tests. All students in the course were asked to provide a unique identifying code on their tests and no other identifying information was provided. The instructor was blind to this code until after all test grades were recorded. Short answer questions were graded on an objective, standardized rubric. This rubric consisted of key points based on in-class notes, which all students in the course had access to during and after each lecture. The final course grades included: 425 total points for aggregated test scores, 3 × 25-point true/false quizzes, 1 × 50-point reaction paper related to reading a peer reviewed article, and 50 subjective attendance points based on the daily class interaction with the instructor.

#### 2.2.2. Cycling PA Outcomes

The study intervention occurred during weeks 4–16 of the semester long course. During lectures, SIT was assigned to sit stationary in a traditional desk for 12 weeks and CYC was instructed to pedal at a low rate of perceived exertion ((RPE), ≤2 out of 10; 1 = no exertion, 10 = maximal effort) during the 50-min lecture, three times a week for 12 weeks. All students were verbally and visually trained on how to utilize an RPE scale in addition to learning about it in their exercise physiology lab, taken concurrently with the lecture course. The minimal cycling RPE was intended to minimize the effect of PA intensity on cognitive control [18,19]. Participants recorded their total cycle pedal time and miles per hour (mph) from the attached bike odometer and their RPE (1–10 scale) for each class period in a notebook stored on the cycle desk.

### 2.3. Data Analysis

Statistical analysis was conducted using SPSS 24.0 (SPSS Inc., Chicago, IL, USA). The independent variable was the cycle desk condition (CYC vs. SIT). The dependent variables were average test scores and final grade; covariates were GPA and PA score. Test results are presented as means ± standard deviations. Independent sample *t*-tests were used to examine differences between test score results (test 1, 2, 3, 4) among CYC vs. SIT. ANCOVAs were used to determine if there were significant academic performance (i.e., total test score average; final course grade) differences between CYC and SIT while controlling for prior GPA and PA score (see Table 1). A chi-square test was used to determine the significance of overall, pooled student perceptions via a post-intervention, researcher developed feedback questionnaire (i.e., CYC and SIT collectively either agreed or disagreed with posed questions using a Likert scale where ‘3’ was neutral and omitted from the analysis). All outcome parameters were tested for normal distribution (Kolmogorov–Smirnov test) and homogeneity of variance (Levene test). Level of significance was set at *p* < 0.05.

## 3. Results

The incorporation of cycle desks into a college classroom setting found stable academic performance. Groups remained evenly matched even after three dropouts from CYC as there were no significant differences in the calculated stratified randomization factor between groups (*p* = 0.46). The study results for academic performance, PA, and a post-intervention survey follow.

### 3.1. Academic Performance

**Figure 2:**
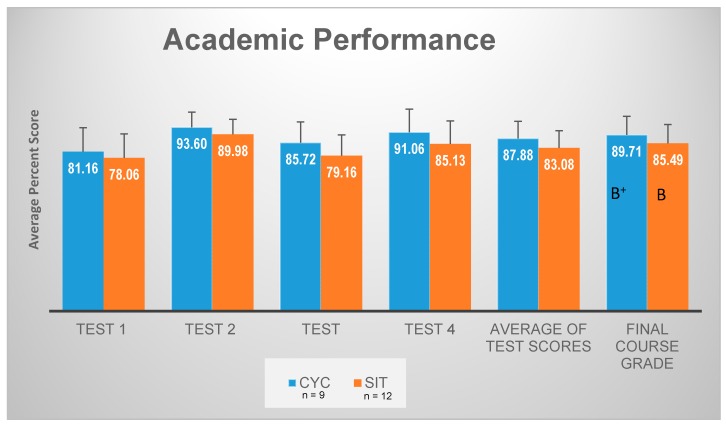
Mean test scores (percent of total points earned) and overall final course grades in CYC (blue) versus SIT (orange) throughout the semester. Standard deviations are represented in the figure by the error bars on each column.

Despite CYC outperforming SIT on all written tests, including overall course grade, these findings were not statistically significant (*p* > 0.05) and are reported in Figure 2. Although no statistical significance between CYC vs. SIT for average of test scores (*p* = 0.431) or final course grades (*p* = 0.185), CYC had higher mean test scores on all tests and a higher overall course letter grade (i.e., B^+^ vs. B, respectively).

### 3.2. Physical Activity

Participants in CYC performed 2550 ± 248 min of cycling during class throughout the 12-week intervention. Average RPE for cycling was 2.3 ± 1.8 out of 10. The course instructor, part of the research team, reported that students did not appear to build up a sweat or be out of breath during lectures, which is consistent with the low RPE reported.

### 3.3. Post-Intervention Survey

The combined post-intervention survey results revealed cycling was not intrusive or distracting (CYC, n = 6; SIT, n = 6) (*p* = 0.004) and incorporating activity into a classroom setting was recommended (CYC, n = 5; SIT, n = 5) (*p* = 0.011). Interestingly, respondents in CYC (n = 6) perceived an increase in daily PA throughout the semester and SIT (n = 5) expressed better awareness of personal PA habits. Note, participant feedback was limited as evidenced by low response rates; despite this, we report the above information while acknowledging the low sample size. Anecdotally, some students enrolled in the exercise physiology course, but not in this study, told the researchers they supported the inclusion of the cycle desks into the classroom. These students also expressed interest in being involved in future cycle desk research.

## 4. Discussion

The purpose of this study was to assess academic performance while using in-class stationary cycle desks during a semester-long college lecture course. This study investigated academic performance between students using cycle desks versus standard or sitting desks to explore the impact on course test grades. Even though the two groups were evenly matched by GPA and PA score), CYC earned, on average, higher test grade values and thus, better overall course grades versus SIT (i.e., B^+^ vs. B, respectively), although they were not statistically different. The results of this study may have been impacted by low participation. Of the 34 students registered for the specific university course, 24 originally wanted to be a part of the study, but after 3 dropouts, 21 students completed the study. We are hopeful that future classrooms with more students will reveal additional information about the feasibility of cycle desks in university lecture settings.

### 4.1. Academic Performance Outcomes

The academic performances of our study participants suggest cycling during a lecture-based course does not impede learning, and perhaps a larger study may prove more beneficial to student learning outcomes. Some literature with relevance to performance, albeit work performance, occurred during a workday and in office settings. Ben-er et al. [20] explored the use of treadmill workstations for a few months in a workplace, with participants walking at low speeds (0–2 mph). They found that overall work performance, quality and quantity of performance, and interactions among co-workers improved as a result of adoption of treadmill workstations. Torbeyns et al. [21] found typing performance and short-term memory were not impacted when people cycled at 30% of maximal workload using watts (Wmax).

Previous research supports that low intensity PA does not lower cognitive performance in both walking [22,23,24,25,26,27,28,29] and cycling programs [12,13,15,30,31,32], conducted in workplace or work simulated settings. Additionally, Barr-Anderson et al. [33] published a systematic review of the literature regarding interventions that integrate short PA sessions (10–15 min) into an organized routine during everyday life in workplace and school settings. They found modest and encouraging consistent results. Their review suggested that brief, even low intensity PA sessions contributed to positive cognitive outcomes. Thus, if these brief PA bouts accumulated over time, such as interrupting sedentary behavior while in a college classroom, promotion of positive academic performance may ensue. In an academic setting, previous investigators indicated brief PA encounters encouraged better focus. Mahar et al. [14] evaluated the effects of a classroom-based PA program on children’s on-task behavior during academic instruction. They found the least on-task students (third and fourth graders) improved on-task behavior by 20% after energizer activities (*p* < 0.001).

### 4.2. Physical Activity Outcomes

Our CYC participants averaged 212.5 min per week in class over the 12-week course. While this contribution to total PA appears small, cycling during class seems to be a convenient way to improve overall college student PA. Although we neglected to measure total daily PA in our participants throughout the semester, a few students made comments on the post-intervention survey that support an increased awareness of PA and inactivity. One student stated “having the bike desk present in class reminded me of how much time I sit daily”. Another student commented, “I wish I had the opportunity to move in my other courses. It would be great if all of my classrooms had the option to cycle during class”. Ben-er et al. [21] found that daily total PA increased as a result of the adoption of treadmill workstations in the workplace, which aligns with the perceptions of some of the participants in our study.

Our research also supports what others have found in workplace settings regarding the contribution of daily PA when it occurs at work. Torbeyns et al. [21] reviewed the research regarding the use of active workstations (standing desks, walking desks, or cycling desks) to fight sedentary behavior in the workplace. Of the 32 studies investigated, 5 were longitudinal studies with school-aged children and 27 were with adults (10 longitudinal studies; 17 non-longitudinal). The general findings supported the use of active workstations because they decreased sitting time, increased energy expenditure, and had positive health outcomes. Although PA intervention protocols varied (i.e., on time, intensity, mode), the outcomes in the particular studies which employed a control group suggested that incorporating some PA during the workday is better than none.

Other research supports the concept that some PA during the workday on a college campus may have positive outcomes. Puig-Ribera et al. [34] assessed the impacts of a workplace web-based intervention (Walk@WorkSpain) on self-reported sitting time, step counts and physical risk factors for chronic disease. Researchers encouraged college campus workers (move, n = 264 vs. control, n = 135) during 19 weeks to decrease occupational sitting time by encouraging progressive incidental movements and short walks during the workday through a web-based system. The intervention group significantly decreased minutes of sedentary time at work from 446 ± 126 to 414 ± 129 with the help of the intervention; while sitting time remained unchanged in the control group (baseline = 404 ± 106; follow-up = 388 ± 120). Also, waist circumference was significantly reduced in the intervention group by 2.1 cm from baseline to follow-up, again supporting that some movement is better than none.

### 4.3. Strengths and Limitations

The present study had a few strengths. To our knowledge, this was the first study conducted in a university classroom during nearly an entire academic semester. Just over 60% of the enrolled students in the course participated in this research. However, the results were limited by sample size. Purchasing more cycle desks and selecting a larger class size (e.g., 400+ students) would provide results that are more reliable. Additionally, we used only one course at a small university so our results are not generalizable to all college students. There are obvious limitations within a college setting given the physical confines of an academic classroom [17] and the length of time of a given course. The feasibility of use and impact of a cycle desk on academic success may need to be measured over a longer period. Our results suggest that incorporating active desks in a university classroom does not interfere with test scores, and may potentially augment academic performance with a concomitant decrease in sedentary behavior (e.g., stationary sitting time).

### 4.4. Future Directions

Our research conclusions necessitate further support by a larger study in diverse college classrooms. A longitudinal design comparing cycle to traditional desks may elucidate a clearer picture of academic performance outcomes. Students from other majors might have different PA and performance outcomes than ones found in our study. Examining the impact of cycle desks on academic performance with other groups of college students such a biology or art majors may reduce potential bias that exercise/sport science majors may have for PA. Measuring other variables, such as the contribution of cycling during class to total daily and weekly PA, and/or students’ feelings and attitude towards the use of cycle desks, are worth exploring further. Other outcomes may be important to consider rather than test scores, such as social interactions, impact on self-esteem, body image, overall wellness, among other variables. Lastly, students may learn the importance of being active in the classroom and continue this behavior in their daily lives, which ultimately benefits society.

## 5. Conclusions

Our aim was to examine the feasibility of using cycle desks in a university classroom and assess academic performance differences between cycle desks versus traditionally sitting in a chair with a table. Based on the post-intervention feedback survey, most participants did not perceive the cycle desks as being disruptive during class and even felt they promoted their awareness of daily PA. Despite limited survey feedback (n = 12), which emphasized participant support for the cycle desks, students (not enrolled in the study, but in the same class) expressed their support for the cycle desks to the instructor at the end of the semester, anecdotally. With no significant differences between in-class chapter test scores, it appears feasible to incorporate low intensity exercise into a classroom lecture venue without negatively altering student academic performance. Perhaps cycling during lecture augments total daily PA, benefits student learning, and/or enhances retention of information. Further research is required with the incorporation of active desks into various classrooms in order to collect more generalizable student perspectives and truly assess whether PA during class time improves or bolsters college student learning. Positive results from a future larger study may encourage a new and innovative way to increase PA in young adults attending college.

## Figures and Tables

**Table 1:** Stratified randomization process for equally-match groups. Participant reported physical activity (PA) score (aerobic duration min/week × intensity (1 = low, 2 = moderate, 3 = high) × frequency (times per week) + estimated resistance training min/week and grade point average (GPA) (mean ± SD) for SIT and CYC groups.

	Weekly PA Score (See Calculation above)	Overall GPA Prior To Study (Based on Highest 4.0 Over Last 5 Semesters)	Stratified Randomization Factor (PA Score × GPA)
CYC n = 9	346.7 ± 208.8	3.3 ± 0.33	1184.4 ± 738.8
SIT n = 12	327.9 ± 191.0	3.3 ± 0.48	1116.0 ± 745.7
